# Assessing the utility of osteoporosis self-assessment tool for Asians in patients undergoing hip surgery

**DOI:** 10.1016/j.afos.2024.01.003

**Published:** 2024-03-02

**Authors:** Keisuke Uemura, Kazuma Takashima, Ryo Higuchi, Sotaro Kono, Hirokazu Mae, Makoto Iwasa, Hirohito Abe, Yuki Maeda, Takayuki Kyo, Takashi Imagama, Wataru Ando, Takashi Sakai, Seiji Okada, Hidetoshi Hamada

**Affiliations:** a Department of Orthopaedic Medical Engineering, Osaka University Graduate School of Medicine, 2-2 Yamadaoka, Suita, Osaka, Japan; b Department of Orthopaedics, Osaka University Graduate School of Medicine, 2-2 Yamadaoka, Suita, Osaka, Japan; c Department of Orthopaedics, National Hospital Organization Osaka National Hospital, 2-1-14, Hoenzaka, Chuou-ku, Osaka, Osaka, Japan; d Department of Orthopaedics, Japan Community Health Care Organization Hoshigaoka Medical Center, 4-8-1, Hoshigaoka, Hirakata, Osaka, Japan; e Department of Orthopaedics, Kansai Medical Hospital, 1-1-7-2, Shinsenri-nishi, Toyonaka, Osaka, Japan; f Department of Orthopaedics, Bell Land General Hospital, 500-3, Higashiyama, Naka-ku, Saka, Osaka, Japan; g Department of Orthopaedics, Yamaguchi University Graduate School of Medicine, 1-1-1, Minami-kogushi, Ube, Yamaguchi, Japan; h Department of Orthopaedics, Kansai Rosai Hospital, 3-1-69, Inabaso, Amagasaki, Hyogo, Japan

**Keywords:** Dual-energy X-ray absorptiometry, Hip surgery, Osteopenia, Osteosarcopenia, Screening

## Abstract

**Objectives:**

Diagnosis and treatment of osteoporosis are instrumental in obtaining good outcomes of hip surgery. Measuring bone mineral density (BMD) using dual-energy X-ray absorptiometry (DXA) is the gold standard for diagnosing osteoporosis. However, due to limited access to DXA, there is a need for a screening tool to identify patients at a higher risk of osteoporosis. We analyzed the potential utility of the Osteoporosis Self-assessment Tool for Asians (OSTA) as a screening tool for osteoporosis.

**Methods:**

A total of 1378 female patients who underwent hip surgery at 8 institutions were analyzed. For each patient, the BMD of the proximal femoral region was measured by DXA (DXA-BMD), and the correlation with OSTA score (as a continuous variable) was assessed. Receiver operating characteristic (ROC) curve analysis was performed to assess the ability of OSTA score to predict osteoporosis. Lastly, the OSTA score was truncated to yield an integer (OSTA index) to clarify the percentage of patients with osteoporosis for each index.

**Results:**

DXA-BMD showed a strong correlation with OSTA (r = 0.683; P < 0.001). On ROC curve analysis, the optimal OSTA score cut-off value of −5.4 was associated with 73.8% sensitivity and 80.9% specificity for diagnosis of osteoporosis (area under the curve: 0.842). A decrease in the OSTA index by 1 unit was associated with a 7.3% increase in the probability of osteoporosis.

**Conclusions:**

OSTA is a potentially useful tool for screening osteoporosis in patients undergoing hip surgery. Our findings may help identify high-risk patients who require further investigation using DXA.

## 1 Introduction

Osteoporosis is characterized by reduced bone mineral density (BMD), which is a risk factor for fracture, particularly in the elderly population. In Japan, an estimated 13 million individuals are affected by osteoporosis, predominantly women [1]. However, many of these patients are undiagnosed and untreated, as the condition often remains asymptomatic until a fragile fracture occurs.

Early diagnosis and treatment of osteoporosis in older people are crucial interventions to help maintain their activities of daily living. Measurement of BMD using dual-energy X-ray absorptiometry (DXA) is the gold standard for diagnosing osteoporosis [2,3]. However, owing to limited access to DXA, several screening tools for osteoporosis have been developed and verified. These include the Simple Calculated Osteoporosis Risk Estimation tool (SCORE) [4], Osteoporosis Risk Assessment Instrument (ORAI) [5], Age, Bulk, One or Never Estrogens (ABONE) [6], and Chinese osteoporosis screening algorithm (COSA) [7]. The Osteoporosis Self-assessment Tool for Asians (OSTA) is a simple screening tool that was developed for Asian females who typically have lower BMD and body weight than their Caucasian counterparts [8]. Use of OSTA requires data on only 2 variables (ie, body weight and age) and the score is calculated using the equation: (body weight − age) × 0.2.

The usefulness of OSTA has been verified in several Asian countries [[8], [9], [10], [11], [12], [13], [14]], including Japan [15]. The results of OSTA were shown to be non-inferior to those of other screening tools that require more parameters [[15], [16], [17]]. The simplicity and effectiveness of OSTA indicate its potential to be used as an initial screening tool. However, OSTA has not been widely adopted, particularly among patients undergoing hip surgery. Consequently, its potential utility as a preliminary screening tool for osteoporosis in this specific context remains uncertain.

The BMD of the proximal femur is important for initial implant fixation and selection of the implant type (eg, cement stem or cementless stem) [18]. However, given that DXA is seldom acquired preoperatively because of the cost and availability, osteoporosis screening using OSTA is clinically in demand. Therefore, we conducted a multicenter study with the following objectives: 1) to assess the relationship between OSTA and BMD of the proximal femur; 2) to establish a simple equation that doctors can easily use for calculating the probability of osteoporosis, and identify patients who require a detailed BMD analysis with DXA.

## 2 Methods

### 2.1 *Participants*

Ethical approval was obtained from the institutional review boards of all institutions participating in this retrospective study (Osaka University IRB number: 21115-2). Informed consent was acquired in the form of opt-out from all patients. The study population consisted of 1378 female patients aged over 50 years who underwent hip surgery at 8 institutions (A–H) (Table 1). The mean ± standard deviation (SD) age, height, weight, and body mass index were 75.5 ± 11.6 years, 151.5 ± 6.8 cm, 51.4 ± 10.6 kg, and 22.3 ± 4.2 kg/m^2^, respectively. There were 633 patients (45.9%) who underwent surgery for proximal femoral fracture (PFF), which was mainly performed in institutions F, G, and H that specialize in trauma surgery (Table 1). Elective hip surgery was performed in institutions A–E which are flagship hospitals, including university hospitals.

**Table 1:** Demographic and clinical characteristics of patients from the 8 participant institutions (named A–H).

Factor	A	B	C	D	E	F	G	H	Overall
Number of cases	192	217	142	111	78	412	135	91	1378
PFF	0 (0%)	0 (0%)	0 (0%)	0 (0%)	0 (0%)	407 (98.8%)	135 (100%)	91 (100%)	633 (45.9%)
Age, yrs	65.2 ± 8.7	67.6 ± 9.1	68.9 ± 8.5	74.6 ± 6.2	68.5 ± 8.4	83.6 ± 8.4	84.2 ± 8.7	84.9 ± 7.6	75.5 ± 11.6
Height, cm	153.8 ± 5.7	153.5 ± 6.3	151.6 ± 5.9	152.4 ± 6.2	152.2 ± 6.0	149.9 ± 7.2	150.1 ± 6.9	149.8 ± 7.4	151.5 ± 6.8
Weight, kg	55.0 ± 9.5	56.2 ± 9.8	55.9 ± 10.0	55.6 ± 10.8	54.5 ± 10.1	47.2 ± 9.5	46.0 ± 8.4	44.2 ± 8.3	51.4 ± 10.6
BMI, kg/m^2^	23.3 ± 3.9	23.9 ± 3.9	24.3 ± 4.1	23.9 ± 4.3	23.5 ± 3.9	21.0 ± 3.9	20.4 ± 3.4	19.7 ± 3.3	22.3 ± 4.2
OSTA index	−2.0 ± 2.7	−2.3 ± 2.9	−2.6 ± 3.0	−3.8 ± 2.7	−2.8 ± 2.8	−7.3 ± 2.9	−7.6 ± 2.9	−8.1 ± 2.5	−4.8 ± 3.8
DXA manufacturer	Hologic	GE	Hologic	Hologic	GE	GE	GE	GE	N.A.
DXA-BMD, g/cm^2^	0.719 ± 0.132	0.715 ± 0.114	0.702 ± 0.127	0.680 ± 0.122	0.705 ± 0.147	0.533 ± 0.109	0.542 ± 0.110	0.522 ± 0.105	0.627 ± 0.147
Osteopenia	87 (45.3%)	105 (48.4%)	72 (50.7%)	58 (52.3%)	31 (39.7%)	68 (16.5%)	36 (26.7%)	16 (17.6%)	473 (34.3%)
Osteoporosis	41 (21.4%)	49 (22.6%)	37 (26.1%)	35 (31.5%)	21 (26.9%)	334 (81.1%)	99 (73.3%)	75 (82.4%)	691 (50.1%)

Values are expressed as mean ± standard deviation or frequency (percentage).

PFF, proximal femoral fracture; BMI, body mass index; OSTA, Osteoporosis Self-assessment Tool for Asians; DXA, dual-energy X-ray absorptiometry; GE, General Electric; NA, not applicable; BMD, bone mineral density.

### 2.2 *DXA acquisition and diagnosis of osteopenia and osteoporosis*

After patient's height and body weight were measured using a scale and/or a tape measure (for measuring height in patients who could not stand up), all patients underwent DXA measurement of the proximal femur before or after the surgery to inform the choice of appropriate surgical implant and initiate treatment for osteoporosis if diagnosed. DXA equipment from 2 different manufacturers was used in the study population (Table 1). Calibration was performed at each institution on each day using a phantom provided by the manufacturer. For patients with hip fractures, DXA was measured at the non-fractured side, and for patients without hip fractures, the operative side (institution A) or the non-operative side (institutions B, E, and F) was measured. In cases where both sides were measured (institutions C and D), the side with the lower BMD was used for the analysis. As BMD values differ between the manufacturers, the value measured in GE's DXA (GE Healthcare Japan, Tokyo, Japan) was converted to that measured in Hologic's DXA (Hologic Japan, Tokyo, Japan) using a previously reported equation [19]. Osteopenia (low bone density) and osteoporosis were diagnosed based on T-scores of −1 and −2.5 at the proximal femoral region (ie, total region), respectively. These T-scores were calculated based on the guidelines of the International Society for Clinical Densitometry [20] with modifications for Japanese. Specifically, T-scores were calculated based on the BMD measurements of young Japanese females (20–29 years, mean BMD in Hologic's DXA: 0.875 ± 0.100 g/cm^2^), which is listed on the guidelines by the Japan Osteoporosis Society [2].

### 2.3 *OSTA and its relationship to osteopenia and osteoporosis*

OSTA was calculated by (body weight − age) × 0.2 and evaluated using 2 methods: one as raw numbers (ie, continuous variable: OSTA score) and the other as OSTA index by rounding up the OSTA score to yield an integer, as described in the original report [8].

The relationship of OSTA between osteopenia and osteoporosis was first assessed by assessing the correlation between OSTA score and DXA-BMD. Then, the diagnostic efficacy of OSTA score for osteopenia/osteoporosis (T-score ≤ −1) and osteoporosis (T-score ≤ −2.5) was assessed using receiver operating characteristic (ROC) curve analysis. Further, to easily predict the prevalence of each patient having osteopenia or osteoporosis, the percentages of patients with osteopenia/osteoporosis and osteoporosis in each index were calculated (eg, (number of patients with osteoporosis with OSTA index at −3)/(number of patients with OSTA index at −3) × 100). The correlation between the OSTA index groups and the percentages of osteopenia/osteoporosis or osteoporosis was assessed.

### 2.4 *Relationship between groups categorized by OSTA and DXA-BMD*

As a sub-analysis, the relationship of groups categorized by OSTA and DXA-BMD was analyzed for patients with PFF (633 cases) and those who underwent elective surgery (745 cases). Specifically, thresholds of −1 and −4 were applied for the OSTA scores because these numbers were originally set to determine the “intermediate risk” and “high risk” of osteoporosis, respectively [8]. For DXA-BMD, thresholds representing a T-score of −1 (0.775 g/cm^2^) and −2.5 (0.625 g/cm^2^) were applied [2]. Using these thresholds, we calculated the sensitivity of the OSTA score and DXA-BMD in predicting PFF. Further, the relationship between groups categorized by OSTA and DXA-BMD was analyzed in patients who underwent elective hip surgery.

### 2.5 *Statistical analysis*

The correlation between the 2 variables was assessed using the Pearson correlation coefficient. A correlation coefficient (r) between 0.6 and 0.8 was considered indicative of a strong correlation [21]. For the ROC curve analysis to diagnose osteopenia/osteoporosis, the area under the curve (AUC), specificity, sensitivity, and cut-off value were quantified. AUC > 0.8 was considered good [22]. Further, as maintaining high sensitivity is important for screening, the optimal cut-off value and specificity to fulfill the sensitivity > 0.9 were also calculated. All statistical analyses were performed using MATLAB v9.10 (MathWorks, Natick, MA, USA), and P-values < 0.05 were considered indicative of statistical significance.

## 3 Results

### 3.1 *Results of DXA-BMD and OSTA*

The mean (±SD) DXA-BMD and OSTA score were 0.627 ± 0.147 g/cm^2^ and −4.8 ± 3.8, respectively. Osteopenia was diagnosed in 473 patients (34.3%) and osteoporosis was diagnosed in 691 patients (50.1%) (Table 1). The prevalence of osteoporosis gradually increased with age (19.1% in the 65–69 year age group; 63.1% in the 80–84 year age group) (Table 2).

**Table 2:** Mean bone mineral density and patients with osteoporosis stratified by age.

Age, yrs	Number of patients	DXA-BMD, g/cm^2^	Osteopenia/Osteoporosis	Osteoporosis
50–54	58	0.762 ± 0.138	29 (50.0%)	11 (19.0%)
55–59	81	0.739 ± 0.126	47 (58.0%)	15 (18.5%)
60–64	131	0.722 ± 0.125	94 (71.8%)	27 (20.6%)
65–69	157	0.707 ± 0.117	124 (79.0%)	30 (19.1%)
70–74	218	0.665 ± 0.127	175 (80.3%)	85 (39.0%)
75–79	165	0.636 ± 0.136	139 (84.2%)	80 (48.5%)
80–84	198	0.587 ± 0.111	187 (94.4%)	125 (63.1%)
Over 85	370	0.507 ± 0.105	369 (99.7%)	317 (85.7%)

Values are expressed as mean ± standard deviation.

DXA, dual-energy X-ray absorptiometry; BMD, bone mineral density.

### 3.2 *Relationship of OSTA with osteopenia and osteoporosis*

**Fig. 1:**
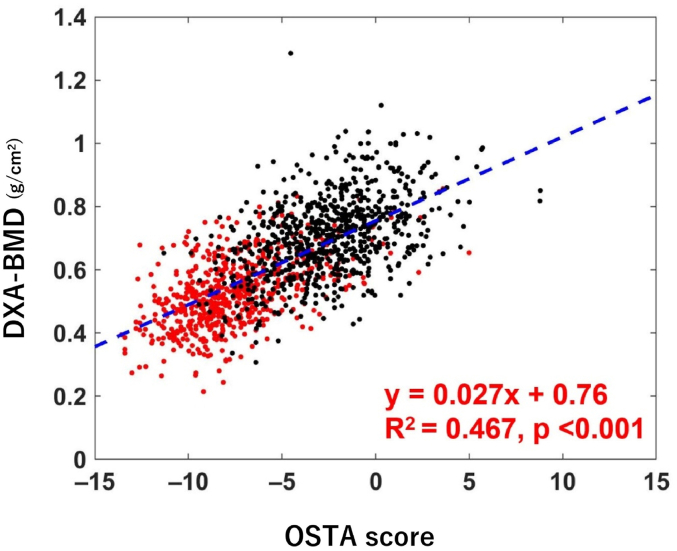
Correlation between Osteoporosis Self-assessment Tool for Asians (OSTA) score and DXA-BMD. Red dots indicate patients with proximal femoral fracture while the black dots indicate the non-fracture cases. The regression line is indicated by the blue dotted line. The regression equation, coefficient of determination, and P-value are indicated in red letters.

**Fig. 2:**
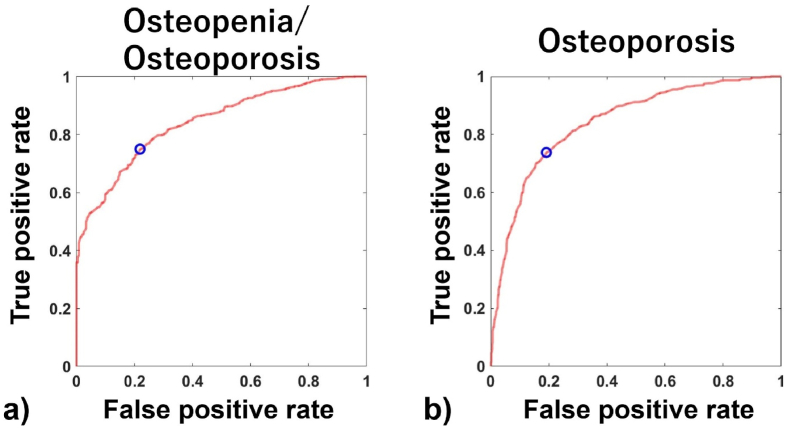
(a) Receiver operating characteristic (ROC) curve for diagnosing osteopenia/osteoporosis (T-score ≤ −1) (a) and osteoporosis (T-score ≤ −2.5) (b) using the Osteoporosis Self-assessment Tool for Asians (OSTA) score. The blue circle indicates the optimal cut-off point.

OSTA score showed a significantly strong correlation with DXA-BMD (correlation coefficient: 0.683; P < 0.001) (Fig. 1). In the ROC curve analysis, the optimal OSTA score cut-off value of −2.76 was associated with 76.8% sensitivity and 75.2% specificity for the diagnosis of osteopenia/osteoporosis (T-score ≤ −1) (AUC: 0.839) (Fig. 2a). For the diagnosis of osteoporosis, the optimal OSTA score cut-off value of −5.4 showed 73.8% sensitivity and 80.9% specificity (AUC: 0.842) (Fig. 2b). Further, with the OSTA score cut-off value of −1 and −4, sensitivity was 96.7% and 83.4%, respectively where the specificity was 30.6% and 66.8%, respectively. To obtain sensitivity of > 0.9, the optimal cut-off value was −2.8 (90.5% sensitivity and 53.4% specificity).

**Fig. 3:**
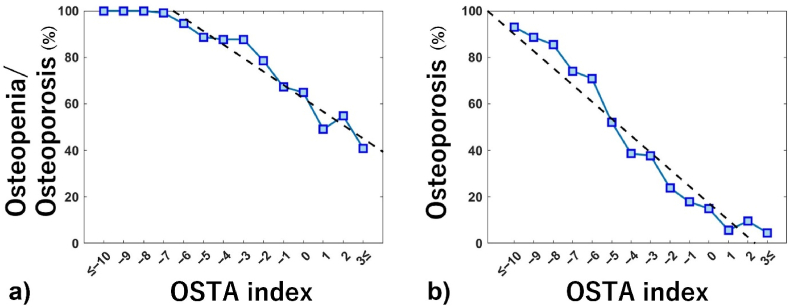
Correlation between OSTA index and the percentages of patients with osteopenia/osteoporosis (T-score ≤ −1) (a) and osteoporosis (T-score ≤ −2.5) (b) at each index. The regression line is indicated by the black dotted line.

On analysis of the OSTA index, all patients with index ≤ −8 demonstrated osteopenia/osteoporosis. The correlation between OSTA index and the percentage of patients with osteopenia/osteoporosis was calculated as (probability in %) = −5.8 × (OSTA index) + 62.4 at OSTA index ≥ −7 (r = −0.97, P < 0.001) (Fig. 3a). For osteoporosis, the probability of patients with osteoporosis showed a linear decrease with increase in the OSTA index. The correlation between the OSTA index and the percentage of patients with osteoporosis was calculated as (probability in %) = −7.3 × (OSTA index) + 17.2 (r = −0.96, P < 0.001), indicating that a decrease in OSTA index by 1 unit was associated with a 7.3% increase in the probability of osteoporosis (P < 0.001) (Fig. 3b).

### 3.3 *Relationship between groups categorized by OSTA and DXA-BMD*

When the 633 patients with PFF were categorized based on OSTA scores and T-scores, the majority of patients (N = 471, 74.4%) were classified as high risk (OSTA scores ≤ 4) and having osteoporosis (T-score ≤ −2.5) (Table 3). The number of patients with PFF diagnosed as osteopenia/osteoporosis (T-score ≤ −1) or osteoporosis based on DXA was 624 (98.6%) and 505 (79.8%), respectively. Conversely, the number of patients with PFF classified as intermediate/high risk (OSTA scores ≤ −1) or high risk (OSTA scores ≤ −4) were 618 (97.6%) and 562 (88.8%), respectively. With the combined use of OSTA scores and T-score, only 4 cases (0.6%) were classified as having low-risk and normal BMD.

**Table 3:** Number of patients categorized by T-score and OSTA score for patients with proximal femoral fracture (upper row) and patients who underwent elective hip surgery (bottom row).

		T-score		
		−1 <	−1∼−2.5	−2.5 ≥
OSTA score	−1 <	4/101	7/98	4/19
	−1∼−4	0/74	26/149	30/62
	−4 ≥	5/30	86/107	471/105

OSTA, Osteoporosis Self-assessment Tool for Asians.

When the 745 patients who underwent elective hip surgery were categorized according to OSTA and T-scores, high risk based on OSTA scores (≤−4) could screen 105 out of 186 patients (56.4%) diagnosed with osteoporosis in DXA (Table 3). Further, intermediate/high risk based on OSTA scores (≤ −1) could screen 167 out of 186 patients (89.8%) diagnosed with osteoporosis in DXA (Table 3).

## 4 Discussion

We applied OSTA to predict osteoporosis in Japanese patients who underwent hip surgery. We observed a strong correlation between the OSTA score and DXA-BMD (r = 0.683). A decrease in OSTA index by 1 was associated with a 7.3% increase in the probability of osteoporosis. Collectively, our results indicate that OSTA can potentially be used as a convenient screening tool for osteoporosis that can help identify patients who may need further BMD assessment using DXA. Further, surgeons can use the results of this study for recommending DXA examination to patients since the likelihood of osteoporosis can be calculated. This information can also be presented to the patient to demonstrate the need for the examination.

### 4.1 *Comparison of our results with previous reports*

Previous studies have demonstrated the effectiveness of OSTA as a screening tool for osteoporosis in Asians. Specifically, the AUC values of OSTA in these studies ranged between 0.62 and 0.87 [[10], [11], [12], [13], [14]], and AUC value in the present study (0.842) is within this range. To the best of our knowledge, only 1 large cohort study by Fujiwara S [15] has validated the use of OSTA for predicting osteoporosis in Japanese women; the optimal cut-off value of −1 in their study was associated with 87% sensitivity and 43% specificity (AUC not reported). In the current study, the OSTA cut-off value of −1 was associated with 96.7% sensitivity and 30.6% specificity, which may be considered comparable. However, the results of our study cannot be directly compared with this study because of the differences with respect to study population (Fujiwara S [15]: long-term epidemiologic study, mean age: 65 years; our study: patients undergoing hip surgery, mean age: 76 years) and region used to diagnose osteoporosis (Fujiwara S [15]: lumbar spine; our study: total proximal femur). The high AUC in our study may be attributable to the older study population and use of BMD of the proximal femur for the diagnosis of osteoporosis; these factors have been reported to positively affect the performance of OSTA [12,13,16].

### 4.2 *Recent tools for screening osteoporosis and future direction of our study*

OSTA is strongly correlated with BMD and could detect osteoporosis, thereby can be considered potentially useful as a screening tool in clinical settings (cut-off value: −2.8 to obtain sensitivity > 0.9). However, the accuracy needs to be further improved because a high false-positive rate may cause unnecessary DXA examination, which is already poorly accessible. While other screening tools such as SCORE [4], ORAI [5], ABONE [6], and COSA [7] may partially serve as the solution, artificial intelligence (AI) technology has recently been applied for evaluating X-rays to screen for osteoporosis. In fact, some studies using AI have shown remarkable accuracy with r > 0.9 in measuring BMD and AUC > 0.9 in detecting osteoporosis from X-ray images [[23], [24], [25]]. However, concerns regarding the robustness and accessibility of AI-developed models have emerged. Further, since our results indicated a correlation of approximately 0.7 and an AUC > 0.8 could be maintained by only using the information of patient's body weight and age, we believe that AI models for screening osteoporosis based on X-rays should clearly indicate how the training and prediction were performed, considering that the “black-box” aspect of AI is a matter of concern. This concern is noteworthy because patient demographics included in AI model training may lead to predicting BMD mainly from the patient demographics rather than from X-ray image itself.

We believe there is scope for improvement in OSTA as a screening tool for osteoporosis using the findings from the hip X-rays. Specifically, parameters such as the Singh index [26], cortical thickness index [27], canal flare index [28], canal-to-calcar ratio [27], and Dorr classification [27] have been widely used by surgeons for estimating the BMD of the proximal femur and determine the surgical implant type to be used. Given that some studies have aimed to use these parameters for initial screening of osteoporosis [[29], [30], [31], [32]], the concomitant use of these radiographic parameters with OSTA should be further evaluated to improve the accuracy of screening osteoporosis [33].

### 4.3 *Sensitivity of OSTA and DXA in predicting PFF*

Both osteopenia/osteoporosis (T-score ≤ −1) and high/intermediate-risk in OSTA (OSTA ≤ −1) showed > 97% sensitivity when applied to patients with PFF. The sensitivity further improved to 99.4% when osteopenia/osteoporosis and intermediate/high-risk in OSTA were combined, which supports the Fracture Risk Assessment (FRAX) Tool to include factors such as age and weight for predicting the 10-year fracture probability [34,35]. As the important role of osteosarcopenia in fractures has gained traction in recent years [36], improving the OSTA score by preventing muscle atrophy and subsequent loss in body weight may play an important role in preventing the occurrence of fractures.

### 4.4 *Application of the results to the general cohort*

One of the key findings of this study is that a single-unit decrease in OSTA was associated with a 7.3% increase in the risk of osteoporosis. Surgeons can use this information to identify patients who require detailed BMD analysis using DXA and can recommend DXA examinations based on the likelihood of osteoporosis. However, caution should be exercised while generalizing our findings to the Japanese population, as only patients who underwent hip surgery were included in this study. At this point, an additional experiment comparing the results with a historical cohort was performed as a post-hoc analysis. Specifically, our results were compared with the historical cohort of Japanese women analyzed in Hologic's DXA (N = 15286) [2]. In the analysis, a significant difference was only observed in the age range of 50–54 years (P = 0.04, unpaired 2-tailed *t*-test, Supplementary Fig. 1). Thus, while further investigation may be necessary, our results may be extrapolatable to the general Japanese cohort.

### 4.5 *Limitations*

Some limitations of this study should be considered while interpreting the results. First, patients included in this study were women aged over 50 years as information on menopause was not available. Thus, the results may change if women who had menopause before 50 years were included. However, the change is expected to be small as the mean age for natural menopause in Japanese women was reported to be around 50 years [37]. Second, the diagnosis of osteoporosis was based on the DXA measurements of the total region of the proximal femur. Results may vary if BMD measurements of the lumbar spine or the neck region were used. However, BMD analysis on the total region is clinically meaningful in patients undergoing hip surgery. Third, while calibration using a phantom was performed in each institution, cross-calibration was not performed in this study. The results may vary if cross-calibration was performed or if DXA from a different manufacturer was used.

## 5 Conclusions

We observed a strong correlation between OSTA score and DXA-BMD. In diagnosing osteopenia/osteoporosis or osteoporosis using the OSTA score, the AUCs were > 0.8. A decrease in the OSTA index by 1 unit was associated with a 7.3% increase in the risk of osteoporosis. OSTA is a potentially useful tool for screening osteoporosis and can help identify patients requiring DXA examination to assist the surgeons in selecting appropriate implant for hip surgery.

## CRediT author statement

**Keisuke Uemura:** Conceptualization, Methodology, Formal analysis and investigation, Project administration, Writing-Original Draft, Funding acquisition, Resources. **Kazuma Takashima:** Writing-Review and editing, Resources. **Ryo Higuchi:** Writing-Review and editing. **Sotaro Kono:** Writing-Review and editing. **Hirokazu Mae:** Writing-Review and editing. **Makoto Iwasa:** Resources. **Hirohito Abe:** Resources. **Yuki Maeda:** Resources. **Takayuki Kyo:** Resources. **Takashi Imagama:** Writing-Review and editing, Resources. **Wataru Ando:** Writing-Review and editing, Resources. **Takashi Sakai:** Writing-Review and editing, Resources. **Seiji Okada:** Supervision. **Hamada Hidetoshi:** Writing-Review and editing, Resources, Supervision.

## Conflicts of interest

The authors declare no competing interests.
