# Supplementary material for: Assessing the utility of osteoporosis self-assessment tool for Asians in patients undergoing hip surgery
Source: Osteoporos Sarcopenia. 2024 Mar 2;10(1):16–21. doi: 10.1016/j.afos.2024.01.003 (PMC11056419; doi:10.1016/j.afos.2024.01.003)
Supplement: Multimedia component 1 [file mmc1.docx]

**
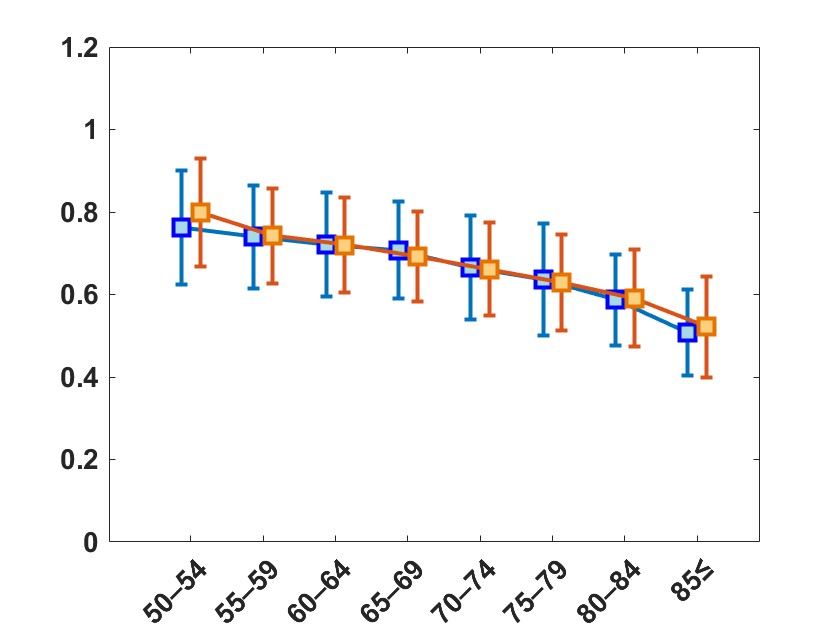
**

**Supplementary Fig. 1** Comparison of bone mineral density measured in dual-energy X-ray absorptiometry (Y-axis) between the patients in this study (blue square) and the previously reported historical control (red square) groups. The only significant difference was observed in the age range (X-axis) of 50-54 years (P = 0.04).
